# Different Coping Patterns among US Graduate and Undergraduate Students during COVID-19 Pandemic: A Machine Learning Approach

**DOI:** 10.3390/ijerph19042430

**Published:** 2022-02-19

**Authors:** Yijun Zhao, Yi Ding, Yangqian Shen, Samuel Failing, Jacqueline Hwang

**Affiliations:** 1 Computer and Information Sciences Department, Fordham University, New York, NY 10023, USA; sfailing@fordham.edu; 2 Graduate School of Education, Fordham University, New York, NY 10023, USA; yding4@fordham.edu (Y.D.); yshen34@fordham.edu (Y.S.); jhwang26@fordham.edu (J.H.)

**Keywords:** machine learning, association rule mining, COVID-19, coping patterns, university students

## Abstract

COVID-19 caused unprecedented disruptions to regular university operations worldwide. Dealing with 100% virtual classrooms and suspension of essential in-person activities resulted in significant stress and anxiety for students coping with isolation, fear, and uncertainties in their academic careers. In this study, we applied a machine learning approach to identify distinct coping patterns between graduate and undergraduate students when facing these challenges. We based our study on a large proprietary dataset collected from 517 students in US professional institutions during an early peak of the pandemic. In particular, we cast our problem under the association rule mining (ARM) framework by introducing a new method to transform survey data into market basket items and customer transactions in which students’ behavioral patterns were analogous to customer purchase patterns. Our experimental results suggested that graduate and undergraduate students adopted different ways of coping that could be attributed to their different maturity levels and lifestyles. Our findings can further serve as a focus of attention (FOA) tool to facilitate customized advising or counseling to address the unique challenges associated with each group that may warrant differentiated interventions.

## 1. Introduction

On 30 January 2020, the World Health Organization declared a global health emergency, and on 11 March 2020, COVID-19 (“coronavirus disease 2019”) was characterized as a pandemic. As the COVID-19 outbreak impacted all aspects of life globally [1] and social distancing policies were implemented to halt virus spread, university students faced particularly unprecedented stressors [2,3,4] as a result of the pandemic [4,5].

Stress occurs when the demands of a situation are perceived to be beyond one’s capabilities to deal with the circumstances [6]. The concept of perceived stress highlights that although people might experience the same event (i.e., the COVID-19 pandemic), their perception dictates their stress appraisal. Thus, an individual’s coping styles might affect how that person perceives stress.

According to Lazarus and Folkman’s (1984) transactional model of stress and coping, personal and situational factors influence how one perceives and evaluates encountered situations. Variables within a person and in the environment (e.g., beliefs, demands, constraints) affect stress appraisal and coping strategy usage, resulting in physiological and emotional reactions [6]. Coping describes the cognitive and behavioral efforts used to manage the internal and external demands of a stressful situation [6,7]. Coping styles and strategies (i.e., confrontive, distancing, self-controlling, seeking social support, accepting responsibility, escape avoidance, planful problem solving, and positive reappraisal) affect the consequences of stressful events, such as the level of anxiety and psychological distress experienced [8,9].

There are different categories of coping strategies. Emotion-focused coping (i.e., reactive) refers to attempts to regulate feelings and emotional responses to the stressor (e.g., anxiety, fear, sadness, anger [7]). Problem-focused (i.e., proactive) coping refers to acting on the stressor, the environment, or oneself to address the problem in an attempt to decrease or eliminate the stress [7]. Problem-focused coping is more effective in controllable stressful situations, but emotion-focused coping is more effective in uncontrollable stressful situations [8,10]. A third type of coping, avoidance-focused coping, refers to cognitions and behaviors aimed at avoiding the stressful situation and reactions to it (i.e., distraction, diversion), which tends to be an initial reaction to stress [8,9,11,12].

Different coping strategies are related to differences in psychological adjustment to stressors [10]. In a study that examined the relationship between stressors and coping in college students during the 2003 SARS epidemic, researchers found that the use of active coping was predictive of life satisfaction, and avoidant coping was predictive of psychological symptoms. Results showed that all types of coping buffered against perceived negative health impacts [10]. In situations of uncontrollable, large-scale stressors, such as SARS 2003 and COVID-19, all coping strategies help to reduce psychological distress.

Despite the positive effects of school closure on reducing the spread of COVID-19, the disruption in learning and the rapid transition to online instruction have inflicted elevated stress, anger, fear, and uncertainty on students across the country. University students, in particular, faced unprecedented and unpredictable difficulties during the pandemic [5,13,14]. In response to the school closure regulations, colleges and universities across the United States abruptly terminated in-person instructions and activities and started transitioning to synchronous and/or asynchronous online learning [5,15]. Campus building accesses such as libraries, gymnasiums, and cafeterias were strictly limited to enhance the safety and security of the university communities. University housing was closed, and students were given only a few days to vacate their dorms on short notice [16,17]. In addition, there were a series of studies examining the overall impact of COVID-19 on college students in other countries, which all provided evidence about challenges encountered by college students [18,19,20,21,22,23,24].

University students also vastly reported psychological distress due to academic difficulties and social isolation. Students were experiencing challenges staying motivated and focused on academics, maintaining emotional well-being, feeling exhausted and generally more cynicism, and decreasing in self-efficacy [13,14,25,26]. Underrepresented minority students also held more negative attitudes towards the transition and experienced more difficulties in submitting online assignments at specific times [14]. Students reported higher anxiety and distress in general, decreased social engagement and connection with peers, professors, and the college community, lack of motivation, and decreased maintenance in healthy habits such as exercising regularly, eating healthy, and sleeping routinely [13,26,27]. Because of their already high level of psychological distress, the psychological effect of social distancing may be aggravated in university students [5].

The current study examined adjustment and coping patterns among undergraduate and graduate students during the COVID-19 outbreak. Specifically, we employed the association rule mining (ARM) algorithm, a machine learning method for discovering interesting relations between variables in observed data [28]. ARM emerged as one of the most popular techniques for pattern recognition in various application domains [29], including COVID-19-related studies [30,31,32]. For instance, Tandan et al. applied ARM to discover symptom patterns of COVID-19 patients. Their study aimed to identify severe COVID-19 cases based on symptom rules from patient management and treatment. Ren et al. investigated potential treatments for COVID-19 based on traditional Chinese medicine (TCM). They used ARM to mine the high-frequency herbs and formulas from ancient prescriptions that were used to treat and prevent viral infection pneumonia diseases for thousands of years. Wu et al. applied ARM to analyze college students’ psychological anxiety and its causes under the global pandemic conditions. Our study applied ARM to explore COVID-19 adjustment and coping patterns among graduate and undergraduate students in US professional institutions. We present the findings and conclusions in the Results and Discussion sections.

### Contribution

This study makes two contributions. First, to the best of our knowledge, there is no existing work that applies ARM to studying the impact of COVID-19 on students’ ways of coping (WOC) behaviors. Our study aims to fill this gap by introducing a new method to transform survey results to market basket items and, subsequently, apply ARM to mine behavioral patterns among university students during an early peak in the pandemic. Furthermore, our data encoding technique can be adopted by other survey-based studies to extract implicit knowledge embedded in the collected survey reports.

Second, although there were studies examining individuals’ psychological distress and changes in cognition and behaviors during the COVID-19 outbreak (e.g., [33,34,35,36], there is a lack of research examining how graduate and undergraduate students differ in their coping behaviors in response to COVID-19. Our study identifies the distinct patterns between the two groups when facing unprecedented uncertainties and provides insights into their idiosyncratic behaviors that are attributed to different maturity levels and lifestyles. Our findings can further serve as a focus of attention (FOA) tool to facilitate customized advising or counseling to address the unique challenges associated with each group, which may require differentiated interventions.

## 2. Materials and Methods

### 2.1. Participants and Procedures

A total of 517 students (242 undergraduates and 275 graduates) at US postsecondary institutions were recruited to participate in this study. They were administered a set of self-report questionnaires through Qualtrics. There were 91 (17.6%) males, 423 (81.8%) females, and 3 (0.6%) students reporting nonbinary gender or other. The age of the participants ranged from 18 to 57 years (M = 24.16, SD = 5.19, N = 517). All participants were provided with informed consent, noting the confidentiality rules and the voluntary participation in this study. The questionnaires took about 10 min to complete. All data were collected from May 2020 to July 2020.

### 2.2. Measures

#### 2.2.1. Demographic Questionnaire

Participants in this study completed a demographic questionnaire to gather information regarding their gender, age, and race. Relevant university and academic information of participants was also collected. Table 1 presents the demographic characteristics of the participants.

#### 2.2.2. Ways of Coping Measures

The Ways of Coping Questionnaire [9] is a self-report measurement initially developed to assess what people do in specific stressful situations, rather than what they typically do or think they should do. It includes 66 items and uses a four-point Likert-scale (from “does not apply or not used” to “used a great deal”) [37]. The WAYS categorizes multiple dimensions of coping into eight subscales. We chose three subscales of interest to analyze the three domains of coping, chosen based on the definition of the WAYS scales and literature description of coping strategy categories [7,9]. The positive reappraisal (emotion-focused; seven-item, α=0.79) subscale measured individuals’ efforts to create positive meaning through focusing on personal growth. The planful problem solving (problem-focused; six-item, α=0.68) subscale measured individuals’ deliberate problem-focused efforts to change the stressful situation. The escape avoidance (avoidance-focused; eight-item, α=0.72) subscale measured participants’ wishful thinking and behavioral efforts to avoid the stressful situation [9,38]. Higher item scores indicated more frequent usage of behaviors of that subscale in coping with COVID-19.

#### 2.2.3. COVID-19 Questionnaire

A 25-item, 5-point Likert-scale (“strongly disagree” to “strongly agree”) was designed for this study to measure the effect of COVID-19 on participants in six subdomains. This COVID-19 measure was an adaptation of an instrument (a 37-item questionnaire) created to measure the mental health index and experiences of university students during the initial outbreak in China [39].

An exploratory factor analysis (EFA; [40,41,42]) was conducted to investigate the factor structure of the COVID-19 questionnaire. The analysis investigated six hypothesized dimensions of emotionality (i.e., social support, academic adjustment, discriminatory impact adjustment, specific regulation reaction, and general regulation reaction) from 25 examined items. The principal axis factoring (PAF) extraction method was used to assess how many factors best represented the dataset, resulting in six factors that met the scree plot criterion [42] and the percentage of variance criterion [43] as shown in Table 2.

**Table 2:** Pattern matrix of factor loadings for 25-item six-factor solution (N = 517).

ID	Question	1	2	3	4	5	6
1	I feel less motivated.	0.82					
2	I procrastinate more now than ever before.	0.77					
3	I feel I am less engaged academically.	0.76					
4	I have not been keeping up to date on my studies.	0.73					
5	I am worried about taking classes and studying online.	0.66					
6	I am worried that I will receive grades that are lower than I originally anticipated.	0.66					
7	I am concerned about staying on top of my academics.	0.62					
8	Below are some descriptions of my personal situation: the coronavirus pandemic is stressful to me; the coronavirus pandemic made it difficult for me to relax; the coronavirus pandemic made me feel like I was consuming a lot of energy; the coronavirus pandemic made it difficult for me to calm down.		0.77				
9	Since the coronavirus outbreak, I have experienced the following emotions: Anxiety, depression, tension, anger, fear, sadness, concern.		0.74				
10	Since the coronavirus outbreak, I am fearful that I will be infected.		0.72				
11	I am worried that my family will get sick with the coronavirus (e.g., siblings, parents, grandparents).		0.56				
12	I feel upset when reading or hearing negative comments about China and Chinese people on the coronavirus.			0.82			
13	I am worried about the discrimination Asians are facing due to coronavirus.			0.80			
14	I am aware of Asians’ experience with discrimination due to the coronavirus.			0.75			
15	I think the regulations imposed as a result of the coronavirus are an overreaction (e.g., school closures, restaurant and bar closures, lockdowns).				0.72		
16R *	I am frustrated that some people are not paying attention to the dangers of coronavirus.				0.70		
17R	I feel relieved that schools are closed and classes have moved online.				0.65		
18R	Below are some descriptions of my personal situation: I think it’s a good idea to be well protected; I want to be well protected; I can be well protected; I know how to protect myself; I have complete control over my protection; I advise others to take precautions.				0.43 **		
19R	I feel supported by my professors and university.					0.78	
20R	I feel supported by my parents.					0.70	
21R	I feel supported by my friends.					0.70	
22R	I am satisfied with the communication from my program/university regarding the coronavirus pandemic.					0.64	
23	I do not agree with strategies related to preventing the spread of the coronavirus (e.g., face masks only need to be worn by people who are sick, not touching my face, social distancing).						0.69
24	It was confusing for me to hear the CDC’s statement that wearing a face mask would not protect me from the coronavirus.						0.66
25	I feel judged wearing a face mask in public.						0.56

* Suffix “R” indicates reversed questions. ** Factor loading <0.5; question removed from the corresponding domain in Table 3.

**Table 3:** Subdomains in COVID-19 questionnaire.

COVID-19 Domain	Item#	Cronbach’s Alpha
Academic Adjustment	1, 2, 3, 4, 5, 6, 7	0.85
Emotionality	8, 9, 10, 11	0.75
Discriminatory Impact Adjustment	12, 13, 14	0.78
Regulation Reaction_General	15, 16R, 17R, 18R	0.63
Social Support	19R, 20R, 21R, 22R	0.69
Regulation Reaction_Specific	23, 24, 25	0.40

Shown in Table 3, the emotionality subscale (four items, α=0.75) measured participants’ emotional thoughts and behaviors towards COVID-19-related stimuli and experiences. The social support subscale (four items, α=0.69) measured participants’ satisfaction with their perceived social support during the COVID-19 outbreak. The academic adjustment subscale (7-item, α=0.85) measured the participants’ perception of their ability to adjust to academic changes due to the COVID-19 pandemic. The discriminatory impact adjustment subscale (three items, α=0.78) measured the participants’ acknowledgment and impact of racism related to COVID-19. The general regulation reaction subscale (three items, α=0.63) measured participants’ agreement with general regulations and restrictions imposed by the government in response to the COVID-19 pandemic. Finally, the specific regulation reaction subscale (three items, α=0.40) measured participants’ agreement with specific regulations and restrictions (i.e., wearing masks) imposed by the government in response to the COVID-19 pandemic. According to Pallant (2016), for scales that have fewer than 10 items, the Cronbach’s alpha levels above 0.50 are sufficient. High scores on each subdomain indicated positive adjustment during the COVID-19 outbreak (i.e., experienced fewer negative emotions; perceived receiving more social support; better adjustment ability to academic changes; less impact of discrimination; and higher agreement with imposed regulations).

To prepare the survey data for the analysis using the machine learning approach, we calculated the average of each participant’s subscale scores and transferred the values to integers following a five-point scale. Specifically, the mean scores between [1, 1.80], [1.81–2.60], [2.61–3.40], [3.41–4.20], and [4.21–5] were recoded to 1, 2, 3, 4, and 5, respectively.

### 2.3. Methods

In this section, we first briefly introduce the association rule mining technique. We then illustrate our method of transforming the survey data into customer-transaction collections under the traditional market basket analysis framework. Lastly, we present the pipeline of our machine learning approach and experimental results.

#### 2.3.1. Association Rule Mining

Association rule mining (ARM) was first introduced for market basket analysis to provide retailers with nontrivial, implicit, and previously unknown information to understand customers’ purchase patterns [44]. The uncovered relationships are represented as “association rules”, such as {Diaper} ⟶ {Beer} [45]. This rule suggests a strong concurrence exists between the sale of diapers and beer in a single transaction (i.e., many customers who bought diapers also bought beer). Consequently, retailers can benefit from this rule to identify new opportunities such as cross-selling or product shelf arrangements. Formally, let:

$$I=\{i_{1},i_{2},\ldots ,i_{d}\}$$

be the set of all distinct items in a market basket data and

$$T=\{t_{1},t_{2},\ldots ,t_{N}\}$$

be the set of all customer transactions, where each transaction $t_{i}$(i=1,2,…,N) contains a subset of items from *I*. We define an itemset *X* to be a collection of items:

$$X=\{x_{1},x_{2},\ldots ,x_{k}\}$$

where $x_{i}\in I$ ∀i∈[1,k] and |A|≤d.

Given a set of transactions *T*, the overarching goal of ARM is to identify non-negligible and strong purchase patterns *r*: $X_{1}⟶X_{2}$ where both $X_{1}$ and $X_{2}$ are itemsets. The non-negligibility is measured by *support*, defined as the proportion of transactions containing both $X_{1}$ and $X_{2}$:

$$support\left(r\right)=\frac{|X_{1}\cup X_{2}|}{\left|T\right|}$$

The implication strength is measured by *confidence*, defined as follows and indicates how often $X_{2}$ is purchased in conjunction with $X_{1}$:

$$confidence\left(r\right)=\frac{s(X_{1}\cup X_{2})}{s(X_{1})}$$

where *s* denotes the support. In practice, two thresholds minsup and minconf are employed to identify strong association rules, which are defined to have their support and confidence above the minsup and minconf, respectively.

In addition to market basket analysis, researchers extended the ARM technique to various real-world applications, including web-usage mining [46], medical diagnosis [47], intrusion detection [48], customer relationship management [49], and bioinformatics [50]. Our study applies ARM to examine distinct coping behaviors between graduate and undergraduate students during the COVID-19 pandemic.

#### 2.3.2. Lift

As illustrated in Section 2.3.1, ARM extracts strong implication rules based on the predefined minsup and minconf thresholds. However, such association rules can be misleading even if they pass both threshold tests. For example, Table 4 summarizes hypothetical commuting and class arrival habits of 1000 college students. The association rule {Biking} ⟶ {On Time}, that is, “students who bike to school tend to be on time for their classes”, is arguably a strong association rule due to its relatively high *support* (15%) and *confidence* (75%). Nevertheless, we observe that the percentage of students on time for their classes in the entire study cohort is 90%. Thus, biking to school has a negative association with being punctual.

To address this issue, we evaluate the validity of *r*: $X_{1}⟶X_{2}$ by computing the *lift* as follows:

$$lift\left(r\right)=\frac{c(X_{1}⟶X_{2})}{s(X_{2})}=\frac{s(X_{1},X_{2})}{s\left(X_{1}\right)s\left(X_{2}\right)}$$

where *s* and *c* denote *support* and *confidence*, respectively. Consequently,

$$lift\left(r\right)\;\left\{\begin{array}{cc} =1 & \mathrm{if}\;X_{1}\;\mathrm{and}\;X_{2}\;\mathrm{are}\;\mathrm{independent}\\ >1 & \mathrm{if}\;X_{1}\;\mathrm{and}\;X_{2}\;\mathrm{are}\;\mathrm{positively}\;\mathrm{correlated}\\ <1 & \mathrm{if}\;X_{1}\;\mathrm{and}\;X_{2}\;\mathrm{are}\;\mathrm{negatively}\;\mathrm{correlated} \end{array}\right.$$

In the above example, *lift* ({Biking} ⟶ {On Time}) = 0.15/(0.2 × 0.9) = 0.8333, indicating a negative correlation. In our study, we require *lift* >1.2 to ensure the validity of the frequent patterns.

#### 2.3.3. Encoding Survey Results as Market Basket Items

One challenge in our study was to render the survey information as market basket items under the conventional ARM framework. To this end, we modeled participants as customers and domain-score pairs as unique items. Consequently, the domain scores associated with each survey report were analogous to items purchased in a customer’s transactions.

Table 5 illustrates our encoding system to transform domain-score pairs into market basket items. Since each domain had fewer than five score categories, we represented each unique domain-score pair by concatenating a domain’s index and a corresponding score as one item. Specifically, the last digit of an item specified the score, and the proceeding string indicated the domain index. For example, “W2-1” represents that the survey taker did not apply the “Planful Problem Solving” coping method. Following this approach, each domain contributed *k* unique items to the market basket where k≤5 is the number of score categories for the domain.

#### 2.3.4. Frequent Pattern Generation

**Figure 1:**
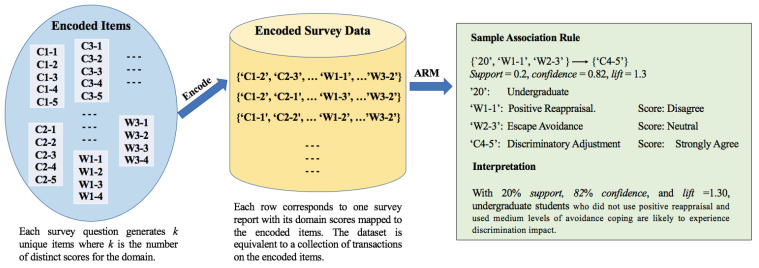
Association rules generation from survey data.

Figure 1 illustrates the pipeline of our frequent pattern generation process. First, all unique items were generated (left component) using the above encoding system. Next, each survey report was modeled as an itemset “purchased” by the survey taker, with each item corresponding to the domain scores of the participant. As a result, the entire dataset (middle component) was equivalent to a set of transactions on the unique encoded market basket items. Finally, we applied association rule mining techniques to extract all strong frequent patterns (right component).

For model implementation, we applied the FP-growth algorithm [51], which is an improved version of the Apriori algorithm [52] in terms of efficiency and scalability. In particular, Apriori requires multiple scans of the dataset to calculate the support of the itemsets, and thus, can be computationally expensive when the dataset is large. FP-growth addresses this issue by storing and reusing information in a tree structure (i.e., FP-tree). Consequently, the algorithm only scans the dataset twice. Once the FP-tree is constructed, FP-growth uses a recursive divide-and-conquer approach to mine the frequent itemsets.

In our experiments, we set the hyperparameters *minsup* and *minconf* (Section 2.3.1) to 20% and 70%, respectively. The identified rules were further filtered by requiring *lift* > 1.20. For instance, a sample association rule produced by our algorithm was as follows:

{‘20’,‘W1-1’,‘W2-3’}⟶{‘C4-5’},support=0.2,confidence=0.82,lift=1.3

where

‘20’ : I am an undergraduate
‘W1-1’ : Positive Reappraisal. Score: Disagree
‘W2-3’ : Escape Avoidance. Score: Neutral
‘C4-5’ : Discriminatory Adjustment. Score: Strongly Agree

The rule indicated that, with 20% *support*, 82% *confidence*, and *lift* = 1.30, undergraduate students who did not use positive reappraisal and used medium levels of avoidance coping were likely to experience discrimination impact.

## 3. Results

Our ARM algorithm produced a total of 14 and 78 strong association rules for the graduate and undergraduate groups, respectively. This large discrepancy suggests that undergraduate students were more consistent in their coping methods in response to the pandemic. One explanation for this outcome is the similarity in lifestyles and social environment of undergraduates compared to that of the graduate students. For example, undergraduate students typically reside in university housing and dine at school cafeterias, while graduate students live off-campus and have a much more diversified social circle. We summarize the major findings for each group in Table 6.

For the graduate group, we observed that students who did not practice planful problem solving and reported exposure to discriminatory language/behaviors during the pandemic were likely to experience strong emotional reactions (G1). Furthermore, students who did not practice positive reappraisal coping methods and reported less social support during the COVID were likely to feel being discriminated against (G2). Lastly, students who demonstrated strong compliance to regulations and experienced intense emotions during the pandemic were likely to report feeling being discriminated against (G3).

For the undergraduate group, we found that individuals who expressed negative emotions tended to use a medium levels of avoidance coping and vice versa (U1, U2, U3). Furthermore, students who did not practice positive reappraisal in combination with other negative patterns (e.g., unsystematic problem-solving, negative emotions, high levels of academic adaptive stress) tended to experience more discriminatory effects (U4, U5, U6, U7, U8). Lastly, individuals who experienced high levels of discrimination in combination with other negative patterns (e.g., reduced social support, regulatory stress) were less likely to use positive reappraisal. (U9, U10).

## 4. Discussion

### 4.1. Coping Patterns in Graduate Students

Coping refers to efforts, both cognitive and behavioral, used to manage demands that put a strain on an individual’s resources [7]. Problem-focused coping strategies attempt to alleviate or eliminate stressful situations by taking control. Emotion-focused coping strategies, such as positive reappraisal, attempt to regulate emotional distress in response to stressful or potentially stressful events. Positive reappraisal relates to the efforts of creating positive meaning by focusing on personal growth. Escape avoidance relates to wishful thinking and behavioral efforts to escape or avoid the stressor [6,7].

Previous studies showed that the use of problem-focused coping styles was associated with lower stress levels [53,54]. This was supported by our findings that individuals who did not practice planful problem solving and had exposure to discriminatory language/behaviors during the pandemic were likely to experience strong emotional reactions (R1). According to Folkman and Lazarus (1984), coping is capable of mediating the emotional outcome. Lazarus (1993) reported that some coping strategies, such as planful problem solving, were associated with changes in emotion from negative to less negative emotions. The absence of problem-focused coping styles might reduce the individual’s focus on changeable elements of the environment (e.g., circumstances that can be changed by taking actions) and increase more negative emotional reactions (e.g., fear, worry, anxiety).

Our findings suggested that individuals who did not practice positive reappraisal coping methods and reported less social support during the pandemic tended to feel more discriminated against. According to Folkman and Lazarus (1988), positive reappraisal was associated with changes in emotions from negative to less negative or positive emotion, while other coping styles such as distancing were associated with more distress. In addition, the COVID-19 pandemic led to many precaution-related practices, such as self-quarantine and reduced social interaction. Thus, social support has become critical to help individuals cope with the stressful situation. Those without sufficient social support might feel more isolated and become more vulnerable to the discriminatory behaviors and attitudes of others.

### 4.2. Coping Patterns in Undergraduate Students

One trend among the undergraduate students in this study was that individuals who did not report exercising positive appraisal, coupled with the presence of other negative patterns (such as less likely to use planful problem solving, experiencing negative emotions, and having a high level of academic adjustment stress) tended to experience more discriminatory impact.

The second trend was that individuals who experienced a high level of discrimination, coupled with other negative patterns (such as less social support, more regulation related stress), were less likely to use positive reappraisal. There is an association between less use of positive reappraisal and more discrimination-related experiences. The display of discriminatory attitudes and behaviors towards a victim is not something that the victim can subjectively change. Thus, the victim’s reaction to such discriminatory attitudes or actions that will further affect the victim’s overall wellbeing [9]. Positive reappraisal coping strategies such as discovering new meanings in life, making changes in one’s self, and finding new sources of hope, help the individuals to focus on positive aspects of self, and divert his/her/their attention from discriminatory attitudes and behaviors. It is plausible that when an individual is in an uncontrollable circumstance such as the COVID-19 pandemic and was exposed to the COVID-19-related discrimination from others, the use of positive reappraisal might be effective in terms of changing one’s subjective feelings, searching for positive aspects in life, and adapting to the environment by adjusting one’s own emotions. Thus, it is reasonable to anticipate that individuals who do not exercise positive appraisal during an uncontrollable circumstance tend to report more discrimination-related impact.

The third trend was that individuals who reported negative emotions tended to use medium levels of avoidance coping. Although Lazarus (1993) indicated that the escape-avoidance coping led to both unsatisfactory outcomes and satisfactory outcomes, our findings suggested that avoidance coping has a strong association with negative emotions. Escape-avoidance coping is often related to behaviors such as hoping for a miracle, sleeping more than usual, refusing to believe what happened and so on. It is typically the first reaction that individuals have towards stress [8,9]. Give that the COVID-19 pandemic is not likely to end soon, such escape-avoidance coping might not lead to actual life changes, and over time, individuals might build up negative emotions.

### 4.3. Age Differences in Coping

Folkman and Lazarus (1980) evaluated age differences in problem-solving and emotion-focused coping in individuals between 40 and 60 years old. It was reported that age differences reflected in coping seemed to be a function of differences in the sources of stress. In general (see data in Table 3), undergraduate students had more association rules (i.e., more consistent patterns) than graduate students. We believe that such discrepancies are partially due to the life circumstance differences between undergraduate students and graduate students; that is, undergraduate student life is more homogeneous, whereas graduate study life tends to be more heterogeneous. In our study, undergraduate students tended to have more homogenous lifestyles, such as residing in dormitories and engaging in more on-campus activities. In contrast, graduate students had more heterogeneous lifestyles, such as whether they lived close to campus or were distance commuters, whether they held full-time or part-time jobs, and whether they had families or were single.

### 4.4. General Patterns Related to the COVID-19 Discrimination

The epicenter of the COVID-19 outbreak was reported to be in Wuhan, Hubei Province, China in November 2019, and then spread internationally [55,56]. Racism was manifested in anti-Chinese and anti-Asian scapegoating for the spread of COVID-19, contributing to reactions of fear, exclusions, microaggressions, and other threatening behaviors towards Asian children and adults. These behaviors were found at the individual level in daily interactions, as well as in the media [57,58]. Reported racialized incidents and acts of prejudice included bullying, harassment, violence, and discrimination. Hate crimes against Asian Americans rose exponentially across the United States in 2020 and continued into 2021 [59,60,61,62,63]. In our study, we used a proprietary dateset consisting of 517 participants. Due to the research purposes of the original data collection, we recruited 105 Asian American and 116 international students, of which a large percentage of them were from China. Thus, a substantial percentage of the participants in this study were either Asian Americans or Asians. It is not surprising to find that many association rules were related to the COVID-19-related racial discrimination.

### 4.5. Limitations

There were several limitations of this study. First, this study relied on self-report questionnaires, which possibly created biases (e.g., participants were answering questions in a socially favorable way). An alternative approach could be to explore individual coping patterns through a qualitative approach, such as in-depth interviews with students enrolled in higher education. Second, we recruited undergraduate and graduate students mainly through social networks of researchers in the field of psychology. Thus, through the outreach to social science undergraduate and graduate students, many of the participating students were females, reflecting the current enrollment trend in psychology or related social science disciplines. Because we used a convenience sampling approach, there was an unbalanced number of males and females in the participant pool. These undergraduate and graduate students represented individuals with high educational attainment. Thus, the findings of this study might not be generalizable to the general population. Third, for the COVID-19 questionnaire, the specific regulation subscale only had three items and Cronbach’s alpha was 0.40, which was lower than 0.50, as suggested by Pallant (2016). Thus, interpretation of this specific subscale warrants cautions. Lastly, this study recruited more female participants than male participants due to the use of convenient samples. Thus, we did not have an even number of participants in terms of females and males. A potential future study could investigate the gender differences in coping patterns among students in higher education.

## 5. Conclusions

In this study, we used association rule mining techniques to study ways of coping among US graduate and undergraduate students during the COVID-19 pandemic. Our findings suggest that the two groups exhibited different coping patterns in practicing escape avoidance, planful problem solving, and positive reappraisal across six adjustment domains. The results further confirm the universal impact of discrimination across both cohorts during the pandemic. Our findings underscore the importance of examining differences in coping patterns in undergraduate and graduate students and the contextual factors related to the individuals’ perceived stress and challenges, including their sources of stress, age differences, and so on. Differentiated coping strategies might be needed to help individuals at different life stages (e.g., young college students versus full-time working graduate students). Despite its limitations, our study can help to identify the unique challenges associated with the two groups, and thus, facilitate targeted advising.

## Figures and Tables

**Table 1:** Demographic characteristics of participants (N = 517).

Variable	Undergraduate (N = 242)		Graduate (N = 275)	
	N	%	N	%
Ethnicity				
White (including Middle Eastern)	99	40.9	136	49.5
Black or African American	5	2.1	7	2.5
Hispanic or Latino	19	7.9	19	6.9
American Indian or Alaska Native	0	0	1	0.4
Asian	101	41.7	101	36.7
Native Hawaiian or other Pacific Islander	1	0.4	1	0.4
Other	17	7	10	3.6
Student Status				
International Student	39	16.1	77	28
Domestic Student	203	83.9	198	72
Major				
STEM	69	28.5	39	14.2
Humanities	8	3.3	39	14.2
Social Science	114	47.1	182	66.2
Medicine or related fields	14	5.8	33	12
Law	0	0	5	1.8
Business	22	9.1	5	1.8
Other	15	6.2	8	2.9
Gender				
Male	50	20.7	41	14.9
Female	192	79.3	232	84.4
Other	0	0	2	0.7

**Table 4:** Sample of students’ lifestyle statistics.

	Commute	Biking	Taking Bus	Total
Class Arrival				
On Time		150	750	900
Tardy		50	50	100
Total		200	800	1000

**Table 5:** Survey results encoding for association rule mining (ARM).

Domain	Description	Score	ARM Item *
COVID-19 Domains			
C1	Emotionality	1. Strongly Disagree 2. Disagree 3. Neutral 4. Agree 5. Strongly Agree	C1-1 C1-2 C1-3 C1-4 C1-5
C2	Social Support	1. Strongly Disagree 2. Disagree 3. Neutral 4. Agree 5. Strongly Agree	C2-1 C2-2 C2-3 C2-4 C2-5
C3	Academic Adjustment	1. Strongly Disagree 2. Disagree 3. Neutral 4. Agree 5. Strongly Agree	C3-1 C3-2 C3-3 C3-4 C3-5
C4	Discriminatory Adjustment	1. Strongly Disagree 2. Disagree 3. Neutral 4. Agree 5. Strongly Agree	C4-1 C4-2 C4-3 C4-4 C4-5
C5	Regulation Reaction - Specific	1. Strongly Disagree 2. Disagree 3. Neutral 4. Agree 5. Strongly Agree	C5-1 C5-2 C5-3 C5-4 C5-5
C6	Regulation Reaction - General	1. Strongly Disagree 2. Disagree 3. Neutral 4. Agree 5. Strongly Agree	C6-1 C6-2 C6-3 C6-4 C6-5
Ways of Coping Domains			
W1	Escape Avoidance	1. Never used 2. Somewhat used 3. Used quite a bit 4. Used a great deal	W1-1 W1-2 W1-3 W1-4
W2	Planful Problem Solving	1. Never used 2. Somewhat used 3. Used quite a bit 4. Used a great deal	W2-1 W2-2 W2-3 W2-4
W3	Positive Reappraisal	1. Never used 2. Somewhat used 3. Used quite a bit 4. Used a great deal	W3-1 W3-2 W3-3 W3-4

* Last digit of an item specifies the score index. The proceeding string indicates the domain.

**Table 6:** Sample association rules for graduate and undergraduate groups.

Index	Encoded Rules	Interpretations
Graduate Group *		
G1	{W2-1, C4-5} → {C1-5}	Students who do not practice planful problem solving and were exposed to discriminatory language/behaviors during the pandemic are likely to experience strong emotional reactions.
G2	{C2-1, W3-1} → { C4-5}	Students who do not use positive reappraisal coping and have less social support during the COVID are likely to experience discrimination.
G3	{C6-2, C1-5} → {C4-5 }	Students who show compliance to regulations and experience intense emotions during COVID are likely to experience discrimination.
Undergraduate Group **		
U1	{W1-3} → {C1-5}	Students who use medium levels of avoidance coping tend to have negative emotions.
U2	{C1-5} → {W1-3}	Students who have negative emotion tend to use medium levels of avoidance coping.
U3	{C4-5, C1-5} → {W1-3}	Students who experience strong discrimination impact and negative emotions are likely to show fair usage of avoidance in coping.
U4	{W3-1} → {C4-5}	Students who do not use positive reappraisal are likely to experience discrimination impact.
U5	{W3-1, W1-1} → {C4-5}	Students who do not use positive reappraisal and use medium levels of avoidance coping are likely to experience discrimination impact.
U6	{W3-1, W2-1} → {C4-5}	Students who do not use positive reappraisal and are less likely to use planful problem solving are likely to experience discrimination impact.
U7	{W3-1, C1-5} → {C4-5}	Students who do not use positive reappraisal and experience negative emotions are likely to experience discrimination.
U8	{C3-5, W3-1} → {C4-5 }	Students who do not use positive reappraisal and experience high academic adjustment stress are likely to experience more discriminatory impact.
U9	{C2-2, C4-5} → {W3-1}	Students who experience less social support and high discrimination during COVID are less likely to use positive reappraisal.
U10	{C6-3, C4-5} → {W3-1}	Students who experience average regulation stress and high discrimination are less likely to use positive reappraisal.

All rules generated with *support* > 0.2, *confidence* > 0.6, and *lift* > 1.3. Please refer to Table 5 for each item code. For example, W2-1 represents “Somewhat used Planful Problem Solving”. * Common item “20” (i.e., graduate students) on the left side of G1–G3 was removed for space efficiency. ** Common item “10” (i.e., undergraduate students) on the left side of U1–U10 was removed for space efficiency

## Data Availability

Data will be provided to qualified investigators upon reasonable request.
